# Indocyanine Green-Conjugated Magnetic Prussian Blue Nanoparticles for Synchronous Photothermal/Photodynamic Tumor Therapy

**DOI:** 10.1007/s40820-018-0227-z

**Published:** 2018-10-25

**Authors:** Peng Xue, Ruihao Yang, Lihong Sun, Qian Li, Lei Zhang, Zhigang Xu, Yuejun Kang

**Affiliations:** 1 Faculty of Materials and Energy, Institute for Clean Energy and Advanced Materials, Southwest University, Chongqing, 400715 People’s Republic of China; 2 Chongqing Engineering Research Center for Micro-Nano Biomedical Materials and Devices, Chongqing, 400715 People’s Republic of China; 3 State Key Laboratory of Silkworm Genome Biology, Southwest University, Chongqing, 400716 People’s Republic of China

**Keywords:** Combination therapy, Prussian blue nanoparticles, Photothermal therapy, Photodynamic therapy, Indocyanine green

## Abstract

Indocyanine green (ICG) is capable of inducing a photothermal effect and the production of cytotoxic reactive oxygen species for cancer therapy. However, the major challenge in applying ICG molecules for antitumor therapy is associated with their instability in aqueous conditions and rapid clearance from blood circulation, which causes insufficient bioavailability at the tumor site. Herein, we conjugated ICG molecules with Prussian blue nanoparticles enclosing a Fe_3_O_4_ nanocore, which was facilitated by cationic polyethyleneimine via electrostatic adsorption. The nanocarrier-loaded ICG formed stable aggregates that enhanced cellular uptake and prevented fluorescence quenching. Moreover, the strong superparamagnetism of the Fe_3_O_4_ core in the obtained nanocomposites further improved cellular internalization of the drugs guided by a localized magnetic field. The therapeutic efficacy of this nanoplatform was evaluated using tumor models established in nude mice, which demonstrated remarkable tumor ablation in vivo due to strong photothermal/photodynamic effects. This study provides promising evidence that this multifunctional nanoagent might function as an efficient mediator for combining photothermal and photodynamic cancer therapy.

**Electronic supplementary material:**

The online version of this article (10.1007/s40820-018-0227-z) contains supplementary material, which is available to authorized users.

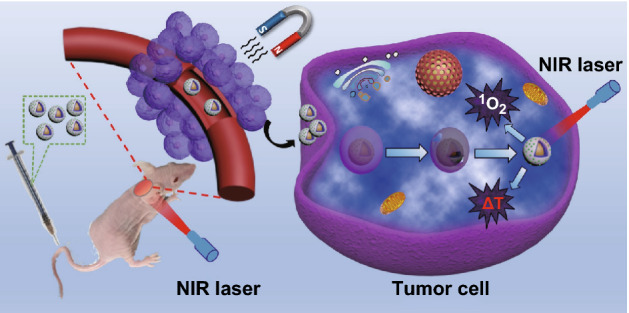

## Highlights

- An efficient photosensitizer, indocyanine green (ICG), was grafted on a nanocarrier via electrostatic adsorption, which effectively resolved poor circulation stability and tumoral bioavailability of free ICG molecules.
- This composite nanoplatform comprised a magnetic core and a photothermal shell, which produced remarkable tumor suppression in vivo under combined photothermal and photodynamic therapy aided by magnetic guidance.

## Introduction

Cancer has become the leading threat to human health due to the increasing rate of incidence and mortality [1, 2]. Many existing therapeutic methods, such as surgery, chemotherapy, radiotherapy, gene therapy, and immunotherapy, present unique advantages and limitations in the treatment of various types of cancers [3, 4]. In recent years, phototherapy activated by light irradiation has drawn increasing attention due to its noninvasive nature during applications [5, 6]. Particularly, photodynamic therapy (PDT) and photothermal therapy (PTT) have shown promising capability with minimal side effects and high selectivity [7, 8]. Photothermal agents that can efficiently induce hyperthermia by converting light irradiation energy are key to achieve effective tumor ablation [9]. Several different classes of functional materials have been developed as photoabsorbers for PTT, including inorganic nanomaterials such as gold nanoparticles (NPs) [10], carbon nanotubes [11], copper chalcogenide [12], and MoS_2_ nanosheets [13], as well as organic nanostructures such as polypyrrole NPs [14], polyaniline NPs [15], and porphysomes [16]. In addition, PDT induces tumor suppression by photosensitizer-mediated generation of reactive oxygen species (ROS) in the tumor environment [17]. Many nanomaterials such as porphyrin [18], gold NPs [19], and quantum dots [20] have been explored as photosensitizers for PDT applications. For the optimal efficacy of phototherapy, it is a rational strategy to develop novel nanocomposites for combining PDT and PTT, thereby taking advantage of both their unique capabilities.

Prussian blue (PB) is a commercial dye approved for the medical management of radiation emergency [21]. A series of PB-based nanoagents have been developed for photothermal applications due to their strong absorbance of near-infrared (NIR) light [22]. Particularly, magnetic PB NPs have been designed and synthesized by enclosing superparamagnetic Fe_3_O_4_ nanocores for magnetically targeted PTT and magnetic resonance imaging [23, 24]. A remarkable internalization of Fe_3_O_4_@PB NPs in tumor cells or tissues was observed under localized magnetic guidance [23]. However, there is a lack of functional groups on PB nanoshells for surface conjugation, which restrains magnetic PB NPs as carriers of therapeutic molecules for cancer treatment. Therefore, there exists a pressing need for new strategies to functionalize PB surfaces and thus combine PTT with other therapeutic modalities (e.g., PDT) [25].

Indocyanine green (ICG) is an approved medical contrast agent for intravenous administration [26, 27]. Remarkable photothermal effects and cytotoxic ROS can be simultaneously produced with ICG under NIR laser irradiation [28]. However, ICG is normally liable to agglomeration and rapid blood clearance, which seriously compromises its capability for PDT or fluorescence imaging applications [29]. To improve the stability of ICG in aqueous conditions, many nanocarriers have been investigated for encapsulating ICG, including calcium phosphate derivatives [30], mesoporous silica NPs [31], and polymer nanocomposites [32]. Because ICG is a negatively charged cyanine dye, it is possible to load ICG by electrostatic adsorption in an aqueous solution [33]. Further, polyethyleneimine (PEI) is a cationic polyelectrolyte that has been widely used as a vector to mediate gene transfection [34]. The ICG ion-paired PEI was previously reported to be encapsulated in a silica matrix [35], in which the fluorescence quenching effect of ICG was significantly reduced owing to the lack of ICG aggregation.

**Fig. 1:**
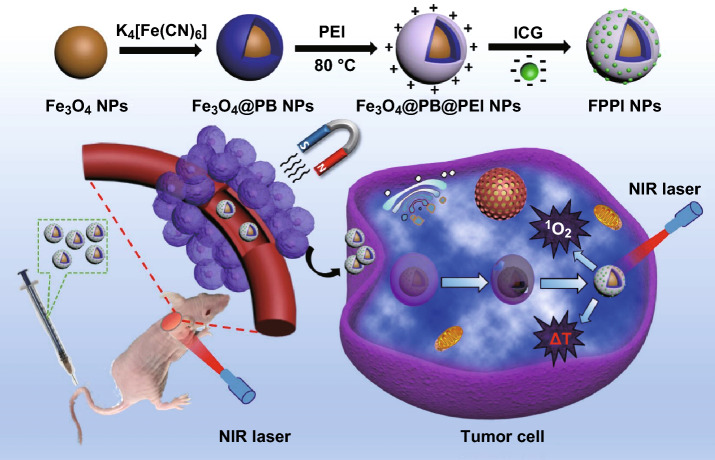
Schematic of the procedure for FPPI NP synthesis and the combination PDT/PTT cancer treatment. Magnetic FPPI NPs accumulate in the tumor region under a local magnetic field, which produces hyperthermia and toxic ROS activated by NIR light irradiation

Based on the above-mentioned properties of PB and ICG, integrating both materials on a single nanoplatform may realize coordinated PDT and PTT activation by NIR irradiation. We hypothesized that PB served as a carrier of ICG to prevent it from degradation and enhanced its stability in aqueous solutions. Herein, we aimed to conjugate ICG with PB NPs for the magnetically targeted combination of PDT and PTT. Specifically, the nanoagent infrastructure was obtained by forming a PB nanoshell surrounding a Fe_3_O_4_ nanocore in situ [23], followed by packaging in PEI to form Fe_3_O_4_@PB/PEI (FPP) NPs. Then, negatively charged ICG was adsorbed onto the positively charged FPP NPs by electrostatic interaction to obtain the final Fe_3_O_4_@PB/PEI/ICG (FPPI) NPs with high drug payload efficiency (Fig. 1). The stability of ICG after being conjugated onto the Fe_3_O_4_@PB/PEI nanocarrier was significantly improved, which benefited its intra-tumoral accumulation. Moreover, the superparamagnetic Fe_3_O_4_ nanocore validated the magnetic guidance to tumor tissues. The PB nanoshell and loaded ICG worked as photoabsorbers for PTT, and the latter also served as a photosensitizer for PDT. The antitumor efficacy of FPPI NPs was investigated using tumor cell lines in vitro and an orthotopic tumor model in vivo.

## Experimental

### Materials

Potassium hexacyanoferrate (II) trihydrate (K_4_Fe(CN)_6_·3H_2_O, 98.5 ~ 102.0%), propidium iodide (PI), iron (II) sulfate heptahydrate (FeSO_4_·7H_2_O, ≥ 99%), fluorescein diacetate (FDA), formalin solution (neutral buffered, 10%), 1, 3-diphenylisobenzofuran (DPBF), and iron (III) chloride hexahydrate (FeCl_3_·6H_2_O, ≥ 98%) were obtained from Sigma-Aldrich (USA). Polyethyleneimine (PEI, Mw = 10,000, 99%) was purchased from Adamas-beta (China). Indocyanine green (ICG), thiazolyl blue tetrazolium bromide (MTT, 98%), and dimethyl sulfoxide (DMSO) were supplied by Aladdin (China). Dulbecco’s modified Eagle medium (DMEM), TrypLE™ Express Enzyme, 4′,6-diamidino-2-phenylindole (DAPI), fetal bovine serum (FBS), penicillin/streptomycin solution, calcein-AM, and phosphate-buffered saline (PBS) were acquired from Thermo Fisher Scientific (USA). The One-Step TUNEL Apoptosis Assay Kit and the mitochondrial membrane potential assay kit with JC-1, and proteinase K were purchased from Beyotime Biotechnology (China). Hydrochloric acid (HCl, 37%) and sodium hydroxide (NaOH) were supplied by Guoyao Group Chemical Reagent Co., Ltd (China). Deionized (DI) water was processed in a Milli-Q water purification system.

### Preparation of FPPI NPs

Fe_3_O_4_ NPs were firstly synthesized following an alkaline precipitation method [36]. Then, a shell-growing process was employed to synthesize Fe_3_O_4_@PB NP_S_ [23]. The detailed procedures for synthesizing these two intermediate products are described in the Supporting Information. Cationic PEI was assembled on the negatively charged Fe_3_O_4_@PB NPs by electrostatic interaction to form Fe_3_O_4_@PB/PEI (FPP) NPs. Briefly, PEI solution (20 mL, 20 mg mL^−1^, pH = 5) was slowly introduced into the Fe_3_O_4_@PB NP dispersion (20 mL) under stirring at 80 °C. After 30 min, the products were purified and collected using a magnet. Subsequent conjugation of negatively charged ICG onto positively charged FPP NPs was performed in a similar manner based on electrostatic interaction. To avoid degradation of ICG, this one-step synthetic procedure was accomplished in the dark. Typically, ICG solution (0.5 mg mL^−1^, 10 mL) was added dropwise into an FPP NP dispersion (20 mL). After 12 h of mixing, FPPI NPs were obtained, purified, and magnetically collected.

### Characterization of FPPI NPs

The physicochemical properties of FPPI NPs were characterized from various perspectives. The UV–Vis–NIR absorbance spectra were obtained using a spectrometer (UV-1800, Shimadzu, Japan). Zetasizer (Nano ZS90, Malvern Instruments, UK) was utilized to measure the hydrodynamic diameter and the surface potential of NPs. X-ray diffraction (XRD) patterns were measured for phase analysis and to determine the crystalline structures of the materials (XRD-7000, Shimadzu, Japan). Fourier transform infrared (FT-IR) spectra of the products from each step were acquired using an FT-IR spectrophotometer (Nicolet 6700, Thermo Scientific, USA). Field-dependent magnetization of solid NPs was measured at 25 °C using a vibrating sample magnetometer (VSM, Lakeshore 7400). The ninhydrin colorimetric assay was employed to quantify amine groups on the surface of Fe_3_O_4_@PB/PEI NPs. Briefly, 500 μL of ninhydrin reagent (0.2% w/v in 0.1 M PBS, pH = 9) was mixed with 200 μL of the Fe_3_O_4_@PB/PEI NP aqueous dispersion (200 µg mL^−1^). The mixture was then incubated in boiling water for 15 min. After cooling down to room temperature and centrifugation at 8000 rpm, the optical absorbance of the supernatants was measured at 570 nm using a microplate reader (SPARK 10 M, TECAN). The amount of conjugated PEI was determined based on a calibration curve derived from ethanolamine as a reference.

### Photothermal and Photodynamic Properties of FPPI NPs

An NIR laser of 808 nm with a power density of 2 W cm^−2^ was used to activate the photothermal and photodynamic effects of the materials. Three milliliters of FPPI NP aqueous dispersions with gradient concentrations (0 ~ 200 μg mL^−1^) were loaded into each transparent quartz vial (capacity: 4 mL) and exposed to NIR irradiation for 10 min, during which the temperature change was recorded in real time using a digital thermometer. For comparison, the photothermal property of intermediate products (200 μg mL^−1^) was also evaluated. To investigate photothermal stability, four cycles of NIR laser irradiation were applied on the FPPI NP dispersion (200 μg mL^−1^), and the resultant temperature variations were recorded in real time during laser irradiation (10 min for each cycle) and cooling processes.

Successful generation of cytotoxic ROS is essential for tumor PDT. As a probe, 1, 3-diphenylisobenzofuran (DPBF) can detect ROS in situ. DPBF was first dissolved in DMSO (20 μL, 1.5 mg mL^−1^) and added to the FPPI NP dispersion (3 mL, 200 μg mL^−1^). The mixture was subject to NIR irradiation, and the UV–Vis absorbance spectrum was recorded using a Shimadzu UV-1800 spectrometer (Shimadzu, Japan). Moreover, 2′,7′-dichlorofluorescein diacetate (DCFH-DA), which is a sensitive fluorescence probe for ROS detection, was utilized to demonstrate intracellular ROS. Briefly, HeLa cells were incubated with free ICG or FPPI NPs (equivalent concentration of ICG: 6 μg mL^−1^) for 4 h and subjected to NIR laser irradiation for 10 min, followed by staining with DCFH-DA solution (10 µM) for 50 min in the dark. After rinsing thrice with DI water, the stained cells were observed under a confocal microscope (LSM 800, Carl Zeiss, Germany).

### In Vitro Effects of FPPI NPs

The therapeutic effect of FPPI NPs on HeLa cells was studied in vitro. In the negative control group, cells were cultured with FPPI NPs (25 μg mL^−1^) for 2 h without laser irradiation or a magnetic field. For the magnetic guidance group, a magnet was placed underneath the culture plate for 2 h. Then, the cultures were exposed to NIR irradiation for 10 min. Afterward, cells were incubated for an additional 1 h before fluorescence staining. Finally, calcein-AM and PI were used to stain viable and dead cells, respectively. To further evaluate apoptosis after various treatments, JC-1 staining was conducted to monitor the change in mitochondrial membrane potential. HeLa cells were treated with ICG or FPPI NPs (at an equivalent ICG concentration of 6 µg mL^−1^) for 4 h and were subjected to NIR laser irradiation for 10 min where applicable. The cells were then incubated with the JC-1 mitochondrial membrane potential (MMP) probe. After 30 min of staining, cells were thoroughly rinsed and fluorescence emission from intracellular JC-1 aggregates and JC-1 monomers was determined using the FITC and Cy3 channels, respectively.

The MTT assay was used to examine the PTT/PDT tumor ablation effect and was carried out in six different groups. Briefly, HeLa cells were cultured for 12 h in a 96-well plate (1 × 10^4^ cells per well) and treated with various agents at gradient concentrations for 2 h. The cells were then subjected to NIR laser irradiation (808 nm, 2 W cm^−2^) for 10 min or a magnetic field for 2 h where applicable. Next, the cells were cultured for another 2 h and subsequently treated with MTT solution (0.5 mg mL^−1^). After an additional 4-h incubation, the old medium was replaced with 200 μL DMSO and gently agitated for 10 min. Finally, the optical absorbance intensity at 490 nm and 630 nm in each well was measured using a microplate reader (SPARK 10 M, Tecan). The relative PTT and PDT effects mediated by FPPI NPs were evaluated using the standard MTT assay in vitro. HeLa cells were seeded in a 96-well plate at 1 × 10^4^ cells per well and cultured at 37 °C overnight. The cells were then exposed to a medium containing FPPI NPs at various concentrations for 2 h. For individual PTT treatment, sodium azide (50 µL, 10 × 10^−6^ M) was added as an ROS scavenger to the culture followed by 10 min of NIR laser irradiation (808 nm, 2 W cm^−2^). For individual PDT treatment, cells were exposed to 10 min of NIR laser irradiation (808 nm, 2 W cm^−2^) at an ambient temperature of 4 °C. For combined PTT/PDT treatment, the cells were exposed to NIR laser irradiation at 37 °C in the absence of sodium azide. After 2 h of further incubation, cell viability was measured using the MTT assay.

### Blood Circulation and In Vivo Biodistribution

Balb/c nude mice (4 ~ 6 weeks of age) were intravenously administered with FPPI NPs (100 µL, equivalent ICG concentration: 8 µg mL^−1^). At pre-designated time points, blood samples were collected from the orbital vein, weighed, and dissolved in chloroazotic acid $\left( {V_{{{\text{HNO}}_{3} }} : \, V_{\text{HCl}} = 1:3} \right)$ for quantification of Fe ions in blood using inductively coupled plasma optical emission spectrometry (ICP-OES). To evaluate the in vivo biodistribution of FPPI NPs, the treated mice were killed post-injection. The major organs and tumors from the mice were collected and lysed in chloroazotic acid $\left( {V_{{{\text{HNO}}_{3} }} : \, V_{\text{HCl}} = 1:3} \right)$. The percentage of NPs retained in tissues was quantified by ICP-OES based on the measurement of the increase in Fe ions content using a calibration curve [37].

### PDT/PTT Efficacy Using FPPI NPs In Vivo

Tumor-bearing nude mice were bred in nine random groups (four in each): (1) saline, (2) NIR laser, (3) free ICG plus laser, (4) PB NPs plus laser, (5) FPP NPs plus laser, (6) FPP NPs plus laser and magnet, (7) FPPI NPs alone, (8) FPPI NPs plus laser, and (9) FPPI NPs plus laser and magnet (100 µL, equivalent ICG concentration: 8 mg mL^−1^). For magnetic targeting, a permanent magnet was placed against the tumor region for 15 min before laser irradiation. After 24 h, the tumor region of every mouse in the laser irradiation groups was exposed to 10 min of NIR laser irradiation, in which the mouse shell temperature was continuously monitored by infrared thermal imaging. During the treatment, the mouse body weight and tumor volume were recorded daily. At day 14, all nude mice were killed to harvest the grown tumors. The apoptotic status of the tumor tissues was then characterized using a One-Step TUNEL Apoptosis Assay Kit. All animal experiments were approved by the Institutional Animal Care and Use Committee (IACUC) of Southwest University and were carried out in compliance with the National Guide for Care and Use of Laboratory Animals. Other general methods can be found in the Supporting Information.

## Results and Discussion

### Synthesis and Characterization

**Fig. 2:**
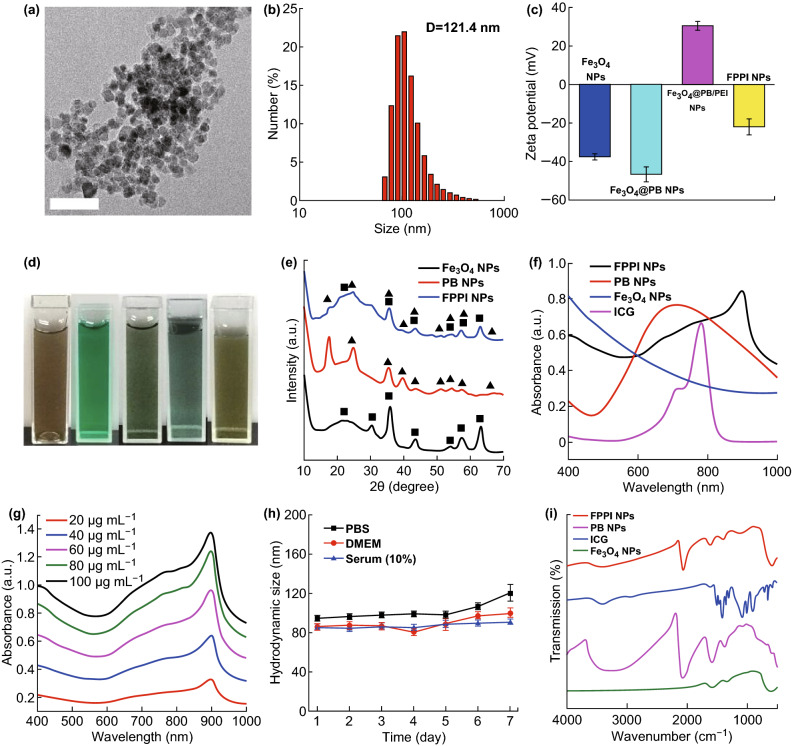
Characterizations of physicochemical properties: **a** high-resolution TEM image of FPPI NPs obtained by electrostatically conjugating ICG onto the surface of magnetic PB NPs (scale bar: 50 nm); **b** hydrodynamic size distribution of FPPI NPs in aqueous suspension measured by the DLS method; **c** zeta potentials of the intermediate and final products; **d** photographs of Fe_3_O_4_ NPs, free ICG, Fe_3_O_4_@PB NPs, Fe_3_O_4_@PB/PEI NPs, and FPPI NPs (from left to right) dispersed in aqueous condition; **e** XRD patterns of Fe_3_O_4_ NPs, PB NPs, and FPPI NPs; **f** Vis–NIR absorbance spectra of Fe_3_O_4_ NPs, PB NPs, ICG, and FPPI NPs dispersed in aqueous solution; **g** Vis–NIR absorbance spectra of FPPI NP dispersions with different concentrations; **h** hydrodynamic size variation of FPPI NPs dispersed in 1 × PBS, DMEM, or 10% FBS over 7 days; **i** FT-IR spectra of Fe_3_O_4_ NPs, PB NPs, ICG, and FPPI NPs in the solid state

Figure 2a shows a transmission electron microscope (TEM) microscopic image of the spherical FPPI NPs, which revealed an average diameter of 12.3 ± 4.8 nm. However, the hydrodynamic size of FPPI NPs in the aqueous dispersion was 121.4 nm, as characterized by the dynamic light scattering (DLS) method (Fig. 2b), and was similar to the previously reported size range of Fe_3_O_4_-based nanoagents for bioimaging applications [38]. For comparison, the dehydrated morphologies of all intermediate products, including Fe_3_O_4_ NPs, Fe_3_O_4_@PB NPs, and Fe_3_O_4_@PB/PEI NPs, were also characterized by TEM (Fig. S1), and their hydrodynamic diameters measured by DLS were 12.9, 75.7, and 85.7 nm, respectively (Fig. S2). Compared to the fully dehydrated state that is required for TEM, these NPs became considerably larger in the aqueous environment, possibly due to the formation of nanoclusters after hydration and slight aggregation of the magnetic NPs [38]. On the other hand, the hydrodynamic size of most FPPI NPs is in the range of 100–200 nm, which has been previously demonstrated as an optimal size range for nanomaterials liable to accumulate in tumor tissues compared to other size ranges (< 100 nm or > 200 nm) [39]. The zeta potential of the intermediate and final products is shown in Fig. 2c. The change in surface potential from negative to positive after PEI coating demonstrated the successful assembly of cationic PEI on Fe_3_O_4_@PB NPs by electrostatic interaction. The ninhydrin-based colorimetric assay is a standard method to quantify the amount of amine groups and was used in this study to quantify the amount of grafted PEI. A strong optical absorbance was observed at 570 nm (purple color in Fig. S3) after incubating Fe_3_O_4_@PB/PEI NPs with ninhydrin reagent, further verifying the successful conjugation of PEI onto the Fe_3_O_4_@PB NPs. The resultant amine content was determined as 3.176 μmol mg^−1^, corresponding to 14.5% PEI (w/w) in Fe_3_O_4_@PB/PEI NPs. Similarly, the zeta potential again became negative on the surface of FPPI NPs, due to the effective conjugation of negatively charged ICG molecules. In addition, all the intermediate and final products could be dispersed into a homogeneous solution (Fig. 2d).

The dispersion of Fe_3_O_4_ NPs exhibited a light brown color, which turned into dark green after forming PB nanoshells followed by PEI coating, while the FPPI NPs displayed a celadon color. XRD characterization was applied to analyze the crystalline structure of Fe_3_O_4_ NPs, Fe_3_O_4_@PB NPs, and FPPI NPs (Fig. 2e). The XRD pattern of Fe_3_O_4_ NPs revealed characteristic peaks at 30.5°, 35.8°, 43.5°, 54°, and 57.5° in accordance with the XRD standard card (JCPDS No. 85-1436), indicating a typical crystalline spine ferrite Fe_3_O_4_ phase. Additionally, the characteristic peaks of 17.5°, 24.7°, 35.5°, 39.7°, 43.5°, 51.2°, 54.3°, and 57.5° were found in PB nanocrystals, corresponding to the XRD standard card (JCPDS No. 73-0687). The resultant FPPI NPs showed all these characteristic peaks of Fe_3_O_4_ and PB nanocrystals without peaks of impurity, suggesting the successful synthesis of FPPI NPs with both crystalline orientations. The Vis–NIR spectra showed strong and broad optical absorbance of FPPI NPs at 700–900 nm (Fig. 2f), suggesting its remarkable potential as a photothermal agent. Particularly, typical absorbance peaks were observed at 780 and 890 nm in accordance with the absorbance peaks of free ICG at 710 and 785 nm. The redshifted absorbance peaks could be due to the formation of ICG dimers or oligomers, also known as J-aggregates [40]. The intensity of optical absorbance was positively correlated with the NPs concentration, indicating their good dispersibility in the aqueous condition (Fig. 2g). A similar optical absorbance effect of FPPI NPs was observed when dispersed in 1 × PBS, DMEM culture medium, and 10% FBS (Fig. S4). Additionally, we monitored the hydrodynamic size variation of FPPI NPs over a prolonged incubation time up to 7 days, where their size did not show notable enlargement until day 6 (Fig. 2h). These characterizations suggested good stability of FPPI NPs, which would benefit their long-term circulation in the body and thereby enhance the bioavailability in tumor regions. In another aspect, the fluorescence emission of FPPI NPs was measured at an excitation wavelength of 765 nm under aqueous conditions. The fluorescence intensity at 795 nm increased linearly with the concentration of FPPI NPs, doubly confirming their good aqueous dispersibility. In addition, FT-IR spectrometry of the key components (Fe_3_O_4_, PB and ICG) was performed to verify the chemical constitution of FPPI NPs (Fig. 2i). Fe_3_O_4_ showed strong absorption near 603 and 1620 cm^−1^ ascribed to the Fe–O and H–O–H vibration, respectively [41]. The PB had characteristic peaks near 2071 and 1625 cm^−1^, corresponding to the bridge vibration of C–N bending in the Fe^2+^–CN–Fe^3+^ and H–O–H stretch, respectively [42]. Additionally, typical peaks at 900–1100 and 1400–1500 cm^−1^ of ICG molecules were attributed to the vinyl stretches and C=C stretches, respectively [43]. The FT-IR spectrum of the final product contained the above characteristic peaks of Fe_3_O_4_, PB, and ICG, which further confirmed the successful synthesis of FPPI nanocomposites. ICG loading capacity and encapsulation efficiency in FPPI NPs were calculated as 7.01% and 69.74%, respectively. Not surprisingly, the high encapsulation efficiency of ICG was attributed to the strong electrostatic interactions between ICG and PEI. Moreover, less than 10% of the ICG molecules were leaked from FPPI NPs after a 12-h incubation in PBS (pH = 7.4 or 6.8), indicating a strong and stable ICG adsorption on FPPI NPs. Additionally, there was no apparent change in the fluorescence spectrum before and after the NIR laser irradiation, verifying the fluorescence stability of the conjugated ICG (Fig. S5).

### Superparamagnetic Property

**Fig. 3:**
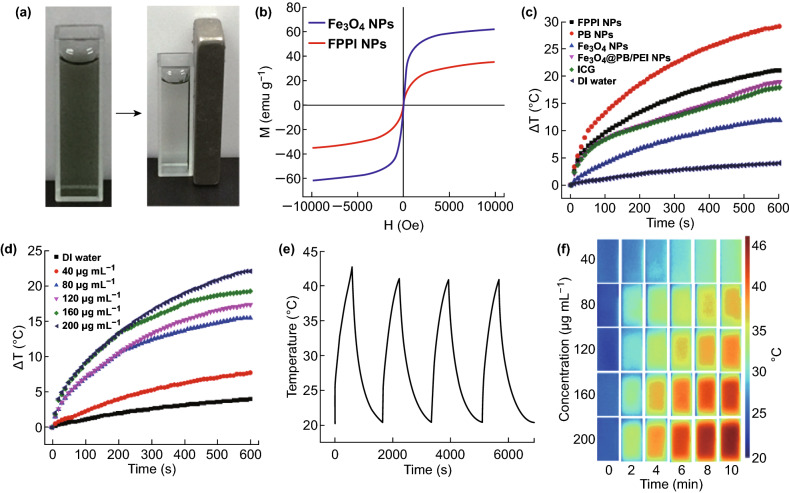
Magnetic and photothermal properties of FPPI NPs: **a** accumulation of FPPI NPs toward a permanent magnet placed against the side wall of a cuvette containing the FPPI NP dispersion (1 mg mL^−1^); **b** comparison of the magnetization response of Fe_3_O_4_ and FPPI NPs at 25 °C using a vibrating sample magnetometer; **c** temperature change in various solutions (200 µg mL^−1^) over 10 min of NIR laser irradiation recorded by a digital thermometer; **d** temperature elevation in FPPI NP aqueous dispersions with various concentrations over 10 min of NIR laser irradiation recorded by a digital thermometer; **e** change in temperature of FPPI NP dispersion under repeated laser irradiation over four consecutive heating–cooling cycles (10 min of irradiation for each cycle); **f** infrared thermal images of cuvettes containing the FPPI NP dispersion to show the temperature change over time under different NP concentrations (NIR laser wavelength: 808 nm; laser power: 2 W cm^−2^)

A magnetic carrier material provides an important means of controlling the spatial distribution of nanodrugs by noninvasive magnetic guidance, thus improving the local bioavailability of therapeutic agents in the tumor region and thereby reducing undesired toxicity to normal tissues. To investigate the magnetic behavior of FPPI NPs, we used a magnet placed next to the side wall of a cuvette containing an aqueous dispersion (1 mg mL^−1^) of FPPI NPs. The NPs rapidly migrated toward the magnet and accumulated against the inner wall within 1 min, and they could be dispersed again homogeneously if the magnet was removed (Fig. 3a). The superparamagnetism of FPPI NPs was found similar to that of Fe_3_O_4_ NPs as characterized by field-dependent magnetization analysis (Fig. 3b). On the other hand, FPPI NPs had a lower saturation magnetization of 35.1 emu g^−1^ compared to the Fe_3_O_4_ NPs (62.2 emu g^−1^). The reduced magnetism of FPPI NPs was due to the compact shielding of nonmagnetic PB and PEI on the Fe_3_O_4_ nanocore.

### Photothermal Property

The FPPI NP dispersion (3 mL, 200 µg mL^−1^) was subject to laser irradiation for 10 min, during which the temperature variation was recorded to evaluate the photothermal property. DI water and intermediate products were also tested for comparison (Fig. 3c). The temperature of the DI water and Fe_3_O_4_ NPs was only increased by 3.5 °C and 4 °C, respectively, implying an insignificant photothermal effect. In contrast, a remarkable temperature elevation was observed in Fe_3_O_4_@PB/PEI NPs, free ICG, FPPI NPs, and PB NPs with increments of 18.9 °C, 17.8 °C, 21 °C, and 29.1 °C, respectively. The FPPI NPs contained the components of the Fe_3_O_4_ nanocore and a PEI polymeric layer, which do not have a photothermal property. Therefore, under the same mass concentration, the laser-induced temperature elevation of pure PB NPs was higher than that of FPPI NPs. The photothermal conversion efficiency of FPPI NPs was calculated to be as high as 51.53% (Fig. S6). These results suggested that the notable photothermal conversion efficiency of FPPI NPs was attributed to the components of the PB nanoshell and conjugated ICG molecules. Additionally, temperature elevation of the FPPI NP dispersion exhibited concentration-dependent and laser power density-dependent profiles (Figs. 3d and S7). The temperature of the FPPI NP dispersion during laser irradiation was also monitored in real time by infrared imaging (Fig. 3f), and the results were in accordance with the reading using a digital thermometer. Such a notable photothermal effect of FPPI NPs may facilitate thermographic imaging in vivo during PTT. The photothermal stability of FPPI NPs was evaluated by applying repetitive laser irradiation and cooling (Fig. 3e). There was an insignificant decrease in the maximal temperature after four cycles of irradiation (10 min for each cycle), indicating the excellent photothermal stability of FPPI NPs.

### Cellular Uptake

**Fig. 4:**
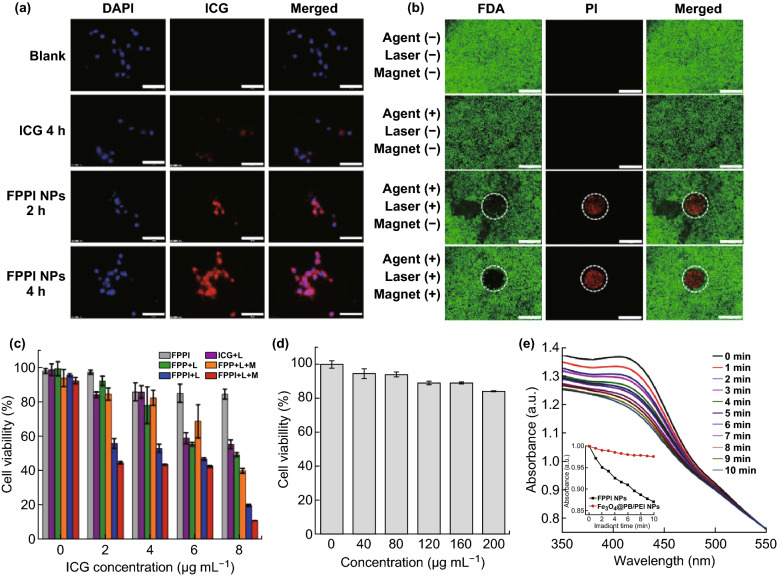
**a** Microscopic analysis of HeLa cells treated with free ICG or FPPI NPs (at the same equivalent ICG concentration) for 2 h and 4 h (scale bars: 100 µm; excitation wavelength: 633 nm). Untreated cells served as the blank control. **b** LIVE/DEAD assays after various treatment combinations, where laser irradiation spots are denoted by dashed circular areas (scale bars: 500 µm). Viable cells and dead cells were stained with green and red fluorescence, respectively. **c** HeLa cell viability after treatment with free ICG, FPP NPs, or FPPI NPs subjected to laser irradiation (“L”) or magnetic field (“M”) where applicable. **d** Impact of FPPI NPs on HUVEC viability after 24 h under various NP concentrations measured by MTT assays. **e** Change in UV–Vis absorbance spectra of FPPI NPs containing DPBF under NIR laser irradiation for 1 ~ 10 min

The intracellular uptake and distribution of FPPI NPs were investigated based on confocal microscopy (Fig. 4a). There was no signal of ICG from the cells in the control group, whereas very weak ICG fluorescence was detected in the cells after treatment with free ICG for 4 h. As mentioned previously, free ICG molecules are unstable in aqueous condition and are prone to aggregation, which impedes their transmembrane conveyance. ICG molecules are also subject to an aggregation-induced fluorescence quenching effect [44]. However, HeLa cells incubated with FPPI NPs showed a prominent ICG fluorescence signal that increased over the time of treatment, indicating the enhanced intracellular uptake of ICG mediated by FPPI NPs. These results provided clear evidence that FPPI NPs were efficient carriers of ICG molecules via endocytosis.

### Antitumor Effect In Vitro

HeLa cells were treated with FPPI NPs (25 µg mL^−1^) and subjected to individual or combination treatment of magnetic field and laser irradiation. After that, LIVE/DEAD viability assay was performed to evaluate the antitumor effect of FPPI NPs (Fig. 4b). Among all the groups, the most significant reduction of viability occurred in HeLa cells incubated with FPPI NPs and subjected to both NIR irradiation and the magnetic field. We also compared the light-induced HeLa cell ablation effect of FPPI NPs, FPP NPs, and free ICG using standard MTT assays (Fig. 4c). Treatment solely with FPPI NPs had an insignificant adverse impact on the cells. However, in the presence of laser exposure, cell viability was decreased notably to 55.1%, 49.17%, and 19.3% after treatment with ICG, FPP NPs, and FPPI NPs (ICG: 8 µg mL^−1^), respectively. Meanwhile, applying a magnetic field further reduced the HeLa cell viability to merely 39.8% and 10.8% in the group of FPP NPs and FPPI NPs, respectively, indicating a magnetically enhanced phototherapeutic effect. To evaluate the relative contribution of PTT and PDT mediated by FPPI NPs, we used sodium azide as a free radical scavenger to remove the intracellular ROS yielded photodynamically, to show the individual PTT effect, whereas another group of cells were incubated at 4 °C during phototherapy to avoid hyperthermia and to show the individual PDT effect. Obviously, the results indicated that applying PTT or PDT individually resulted in an inferior tumor destruction effect compared to the combined PTT/PDT treatment (Fig. S8). Moreover, the IC_50_ values of FPPI NPs with individual PTT, PDT, and combined PTT/PDT were 13.74, 12.79, and 5.2 µg mL^−1^ (equivalent ICG concentration), respectively. To evaluate the potential synergism of PDT and PTT, the combination index (CI) could be calculated as Eq. 1:

1 $${\text{CI}} = D_{\text{A}} /{\text{IC}}_{{50({\text{A}})}} + D_{B} /{\text{IC}}_{{50({\text{B}})}} ,$$

where *D*_A_ and *D*_B_ represent the concentrations of FPPI NPs at IC_50_ based on combined PTT/PDT, and IC_50(A)_ and IC_50(B)_ represent the IC_50_ values for individually applied PTT and PDT, respectively [45]. The CI of these two treatment modalities was calculated as 0.79, indicating a distinct synergistic effect (CI < 1) [46]. The biocompatibility of FPPI NPs was evaluated on HUVECs using standard MTT assays (Fig. 4d). The viability of HUVECs was maintained above 80% under a wide range of dosages (40 ~ 200 µg mL^−1^), suggesting good biocompatibility of FPPI NPs.

### Photodynamic Property

**Fig. 5:**
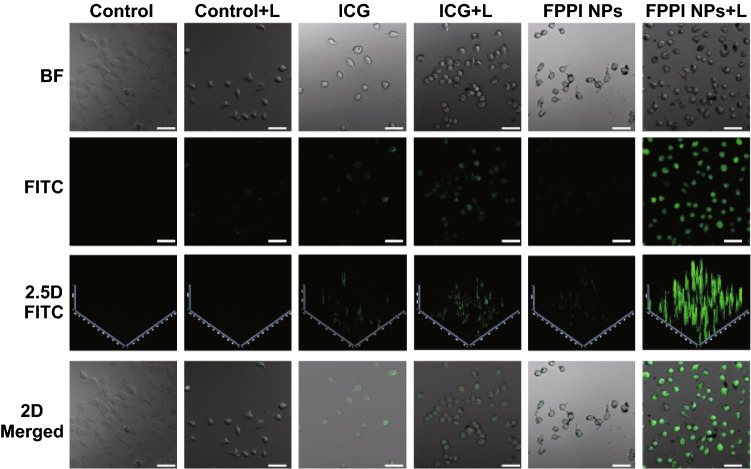
Detection of intracellular ROS using DCFH-DA probes (FITC channel) after HeLa cells were treated with free ICG and FPPI NPs (at an equivalent ICG concentration of 6 µg mL^−1^) for 4 h and subjected to NIR irradiation (“L”) for 10 min where applicable (scale bars: 50 µm)

ICG-mediated generation of ROS is a prerequisite for PDT applications. To further evaluate the PDT capability of FPPI NPs, DPBF and DCFH-DA probes were utilized for ROS detection in aqueous solution and in tumor cells, respectively [47]. DPBF irreversibly binds with reactive ROS, leading to optical absorption decay at 417 nm during photosensitization. Therefore, the rate of DPBF absorption decay is highly related to the ROS yield during the photodynamic process, which can be measured by UV–Vis–NIR spectrometry. Herein, the FPPI NP dispersion containing DPBF was exposed to NIR irradiation for up to 10 min, and the optical absorbance intensity at 417 nm decreased gradually over the time of irradiation (Fig. 4e). Moreover, the absorption decay corresponding to FPPI NPs was comparable with that of free ICG, but was much faster than that of Fe_3_O_4_@PB/PEI NPs (Fig. S9), indicating a higher rate of ROS generation using FPPI NPs. Furthermore, ROS can oxidize DCFH-DA into a green fluorescent compound (DCF), which is also used as a marker for intracellular ROS. Therefore, we introduced DCFH-DA to treat HeLa cells and then examined green fluorescence from the oxidation product of DCF by fluorescence microscopy (Fig. 5). In the absence of laser irradiation, there was a negligible level of ROS generation in the cytoplasm. In contrast, green fluorescence appeared in the ICG-treated and FPPI-treated cells, showing the amount of ROS induced by the photodynamic effect. It was obvious that FPPI NP-mediated photosensitization generated remarkably higher levels of ROS compared to free ICG.

### Apoptosis

**Fig. 6:**
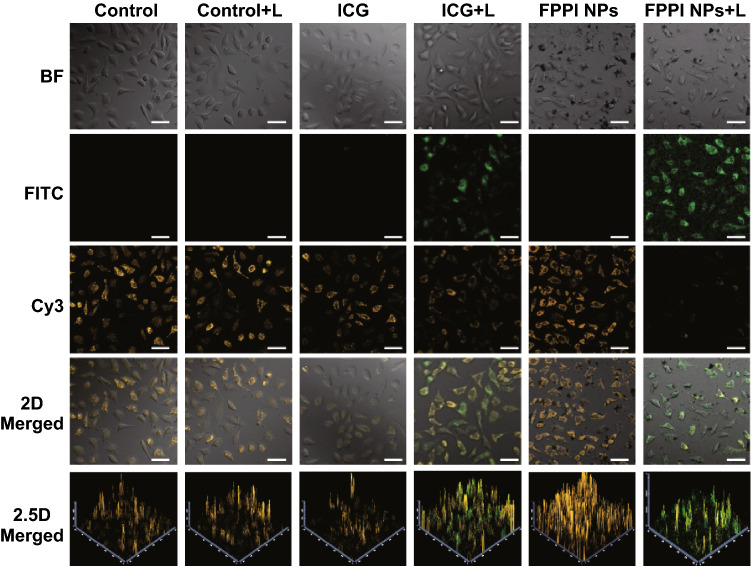
Change in mitochondrial membrane potential of HeLa cells after treatment with ICG or FPPI NPs (at an equivalent ICG concentration of 6 µg mL^−1^) for 4 h and NIR laser irradiation (“L”) for 10 min where applicable. After standard JC-1 staining for 30 min, the apoptosis status was determined by fluorescence images captured via the Cy3 channel for JC-1 aggregates and FITC channel for JC-1 monomers (scale bars: 50 µm)

The decrease in mitochondrial membrane potential (MMP) was found as an indicator of early cell apoptosis [48]. As a fluorescent cationic dye, JC-1 was used to evaluate the altered MMP in HeLa cells. For normal mitochondria, high MMP enables JC-1 to form aggregates with orange fluorescence emission. When mitochondria are damaged, the decreased MMP results in disaggregation of JC-1 into monomers with green fluorescence. The change in JC-1 fluorescence from orange to green indicates the cell fate shifting to apoptosis. The cell groups treated with FPPI NPs in the absence of irradiation exhibited strong orange fluorescence similar to normal cells (Fig. 6), indicating the typical MMP of normal mitochondria. However, remarkable green fluorescence was observed in cells treated with FPPI NPs under laser irradiation, suggesting the decreased MMP of damaged mitochondria. We speculated that FPPI NP-mediated local hyperthermia and ROS production under laser irradiation both contributed to the mitochondrial dysfunction and subsequently induced apoptosis.

### Pharmacokinetics and Biodistribution In Vivo

Effective accumulation of NPs in the tumor region through an enhanced permeability and retention (EPR) effect is a prerequisite for NP-mediated multimodal cancer therapy. The pharmacokinetic behavior of FPPI NPs in vivo was evaluated for understanding their excretion process (Fig. S10a). Fitting with a two-compartment model exhibited a half-life of 1.528 and 9.539 h for the first and second phases, respectively. Then, major organs of mice were collected at 2, 12, 24, and 48 h to evaluate the biodistribution of FPPI NPs (Fig. S10b). The results revealed a large amount of FPPI NPs accumulated in the reticuloendothelial system (RES), including the liver and spleen. A relatively higher retention of FPPI NPs in tumor tissues was also found (12.47% ID g^−1^) at 24 h post-injection, indicating the efficient accumulation of NPs aided by the EPR effect. Moreover, small quantities of NPs were found in the kidneys, suggesting a potential excretion pathway through the renal system.

### Antitumor Efficacy In Vivo

**Fig. 7:**
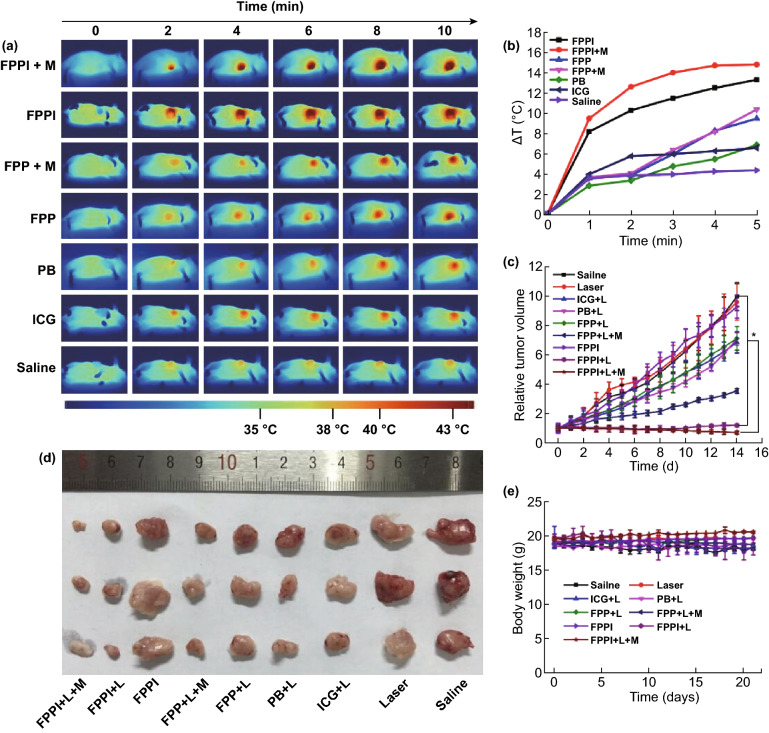
**a** Changes in mouse body shell temperature under NIR laser irradiation over 10 min measured by infrared imaging at 24 h post-injection; **b** local peak temperature elevation in the tumor region during laser irradiation corresponding to **a**; **c** change in tumor volume over time in each group after laser-activated treatment; **d** photographs of the excised tumors in different groups at day 14 post-injection; **e** variation of mouse body weight over time in each group after various treatments

A 4T1 tumor model was established in nude mice to study the therapeutic effect of combination PTT/PDT in vivo. The shell temperature of tumor-bearing mice during NIR laser irradiation was monitored using an infrared imaging system. Basically, the temperature at the tumor site in all groups gradually increased over the time of laser irradiation (Fig. 7a, b). Mice treated with FPPI NPs exhibited the highest temperature of 51.7 °C at the tumor site within 10 min of irradiation, which was much higher than that in mice treated with free ICG (43.5 °C) or PB NPs (43.8 °C). Such strong hyperthermia was ascribed to the efficient photothermal conversion capability of the PB component and improved ICG internalization. After laser-activated treatments, the tumor volume and mouse body weight were recorded daily to evaluate the tumor suppression effect. In the groups treated with FPPI NPs alone, PB NPs, and free ICG (plus NIR exposure), the tumor volume rapidly increased 9.28-, 7.07-, and 6.84-fold at day 14 as compared to the initial tumor size, respectively, suggesting that PB or free ICG-mediated PTT/PDT had a limited tumor inhibition effect (Fig. 7c). In contrast, the relative tumor volume was gradually reduced to 1.19 in the group subjected to FPPI NPs plus laser irradiation with an impressive tumor growth inhibition (TGI) rate of 88.1%. Notably, the highest TGI of 93.0% was achieved in the group of FPPI NPs with magnetic guidance as a result of the increased accumulation of therapeutic agents in the tumor. The size and weight of excised tumors further demonstrated the same effect as revealed by the tumor volume data (Figs. 7d, S11). Moreover, we did not find a significant loss of mouse body weight in all the examined groups during the treatments, indicating a low systemic toxicity of the applied formulations (Fig. 7e). These results demonstrate a strong therapeutic effect of combining PTT and PDT via FPPI NPs, which was consistent with the findings of in vitro experiments.

**Fig. 8:**
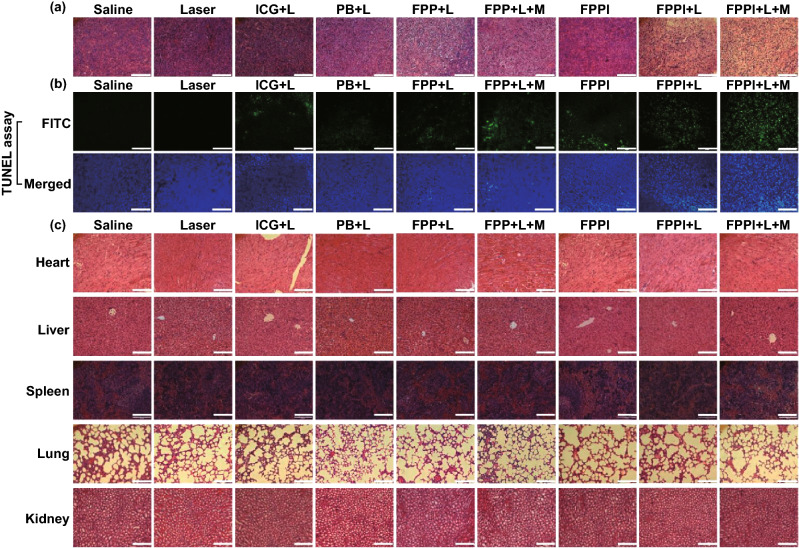
Histological analyses of the excised tumors at day 14 post-injection by **a** H&E staining and **b** TUNEL assays (scale bars: 100 µm); **c** histological analysis of major organs by H&E staining at day 14 post-injection (scale bars: 200 µm). “L” and “M” denote laser irradiation and magnetic guidance, respectively

To examine the deterioration of tumor cells after combination PTT and PDT, H&E staining of tumor histological sections was performed at day 14. Compared to all the control groups, obvious karyorrhexis, pyknosis, and karyolysis occurred in the group treated with FPPI NPs and subject to both laser irradiation and magnetic field, suggesting severe tumor cell destruction (Fig. 8a). In addition, the sectioned tumor tissues were analyzed by TUNEL staining (Fig. 8b), which presented the status of apoptosis. Similarly, the FPPI NP-mediated combination treatment under magnetic guidance produced the strongest green fluorescence, implying the most significant apoptosis among all groups. These assays further confirmed the efficient tumor inhibitory effect of FPPI NPs activated by NIR irradiation and magnetic guidance. There are many prior reports on versatile nanomaterials to realize synergistic photothermal and photodynamic therapy of cancer simultaneously. For example, in 2014, Chen’s group reported a type of photosensitizer-loaded micelles integrated with cyanine dye for synergistically achieving PTT/PDT with a TGI rate of ~ 90% [49]. In 2016, Zheng’s group reported iRGD-modified ICG liposomes for PTT/PDT against breast tumors with a TGI up to ~ 98% [50]. In 2018, Shen’s group reported the assembly of iron oxide carbon dot NPs conjugated with black phosphorus quantum dots and realized a TGI of 98.8% [51]. Compared with these functional antitumor nanomaterials, the FPPI NPs developed in the present work demonstrated comparable tumor inhibition performance (TGI = 93%), implying a promising potential for cancer therapeutics.

### Biocompatibility Evaluation

The biocompatibility of FPPI NPs was evaluated in terms of hemocompatibility and histocompatibility. To assess the biocompatibility of FPPI NPs in blood, the potential hemolysis of RBCs was characterized after co-incubation with FPPI NPs at various concentrations (Fig. S12). For comparison, DI water that caused release of hemoglobin from lysed RBCs worked as a positive control, whereas 1 × PBS that maintained the integrity of RBCs worked as a negative control. The results showed that the hemolysis rate of RBCs was positively correlated with the concentration of FPPI NPs and incubation time. Meanwhile, the hemolysis rate was only 6.1% when the NP concentration was as high as 200 µg mL^−1^ after 4 h of co-incubation, indicating acceptable hemocompatibility of FPPI NPs. Histocompatibility was studied based on live mouse models intravenously administered with FPPI NPs under various treatments. After two weeks, major organs were excised from the killed mice and examined by histopathological analysis (Fig. 8c). There was no apparent inflammation or lesion observed at the cellular or tissue level in the ICG-treated or FPPI-treated mice. Routine hematology studies after administration of nanoagents were also performed to obtain the complete count of critical blood components (Fig. S13). The data showed an insignificant difference in terms of the listed hematological or biochemical parameters in the treated mice compared to the control, indicating the negligible long-term systemic toxicity of FPPI NPs after intravenous administration.

## Conclusions

In summary, a multifunctional therapeutic nanoplatform, abbreviated as FPPI NPs, for combining photothermal and photodynamic cancer therapy was developed by conjugating indocyanine green (ICG) with magnetic Prussian blue NPs through cationic polyethyleneimine. The adsorbed ICG molecules on the surface of the Prussian blue nanoshell formed stable aggregates, facilitating cellular uptake of the drugs. The therapeutic efficacy of this nanoplatform was investigated systematically based on tumor cell lines and orthotopic tumor models. Infrared thermo-imaging revealed that FPPI NPs were able to accumulate at the tumor site in nude mice after injecting the drugs intravenously. When the tumor cells or tissues were subjected to NIR laser irradiation, the internalized FPPI NPs produced strong photothermal and photodynamic effects, which could be further enhanced by magnetic targeting and significantly suppressed tumor growth by inducing apoptosis. In addition, the FPPI NPs exhibited excellent hemocompatibility and histocompatibility with minimal systemic toxicity on the mouse models, indicating a promising potential for combination cancer therapy.

## Electronic supplementary material

Below is the link to the electronic supplementary material.

Supplementary material 1 (PDF 1364 kb)
