# Supplementary material for: Indocyanine Green-Conjugated Magnetic Prussian Blue Nanoparticles for Synchronous Photothermal/Photodynamic Tumor Therapy
Source: Nanomicro Lett. 2018 Oct 25;10(4):74. doi: 10.1007/s40820-018-0227-z (PMC6208784; doi:10.1007/s40820-018-0227-z)
Supplement: Supplementary file 1 — Supplementary material 1 (PDF 1364 kb) [file 40820_2018_227_MOESM1_ESM.pdf]

Supporting Information for

Indocyanine Green-Conjugated Magnetic Prussian Blue Nanoparticles for Synchronous Photothermal/Photodynamic Tumor Therapy

Peng Xue[1, 2, *, #] Ruihao Yang[1, 2, #], Lihong Sun[1, 2], Qian Li[1, 2], Lei Zhang[3], Zhigang Xu[1, 2], Yuejun Kang[1, 2, *]

[1]Institute for Clean Energy and Advanced Materials, Faculty of Materials and Energy, Southwest University, Chongqing 400715, People's Republic of China

[2]Chongqing Engineering Research Center for Micro-Nano Biomedical Materials and Devices, Chongqing 400715, People's Republic of China

[3]State Key Laboratory of Silkworm Genome Biology, Southwest University, Chongqing 400716, People's Republic of China

*Corresponding authors. xuepeng@swu.edu.cn (P. Xue); yjkang@swu.edu.cn (Y. Kang)

# P. Xue and R. Yang contributed equally to this work.

S1 Supplementary Methods

S1.1 Synthesis of Fe_3O_4 NPs

Firstly, $FeCl_3·6H_2O$ and $FeSO_4·7H_2O$ (molar ratio = 2:1) were added into 5 mL of DI water. Then, 170 µL hydrochloric acid (37%) was introduced into the mixture. The obtained reactant was added dropwise into 50 mL NaOH aqueous solution (1.5 M) under vigorous mechanical stirring at 80 °C. As-synthesized magnetic Fe_3O_4 NPs were washed with DI water three times and collected using an external magnet.

S1.2 Synthesis of Fe_3O_4@PB NPs

Briefly, 10 mL of Fe_3O_4 NP dispersion (1 mg mL^{-1}) were mixed with 20 mL of $K_4[Fe(CN)_6]$ solution (50 mM, pH = 3). The mixture was stirred for 15 min until the color changed from brown to dark green. Next, 1 mL of $FeCl_3$ solution (50 mM) was added dropwise into the mixture. The color of the mixture gradually became light green. The products were rinsed with DI water five times and separated using a magnet.

S1.3 Calculation of the Photothermal Conversion Efficiency [S1]

The total energy balance between the input and dissipation for the system is presented as Eq. S1:

$$\sum_i M_i C_i \frac{dT}{dt} = Q_{NP} + Q_{sys} - Q_{out} \quad (S1)$$

where M and C donates the mass and heat capacity of water, respectively; T represents the medium temperature; Q_{NP} is the energy absorbed by NPs; Q_{sys} donates the energy from the pure water system; Q_{out} is heat dissipation of the system.

The heat absorbed by FPPI NPs can be calculated as Eq. S2:

$$Q_{NP} = I(1 - 10^{-A_{808}})\eta \qquad \text{(S2)}$$

where I is the power of NIR laser, η indicates the photothermal conversion efficiency, and A_{808} donates the absorbance of FPPI NPs at 808 nm.

Heat dissipation is linear to the system temperature, shown as Eq. S3:

$$Q_{out} = hS(T - T_{surr}) \qquad \text{(S3)}$$

where h is the heat transfer coefficient, S is surface area of the container, and T_{surr} is the ambient temperature.

After reaching a steady state temperature (T_{max}), the input and output of heat are in equilibrium.

$$Q_{NP} + Q_{sys} = Q_{out} = hS(T_{max} - T_{surr}) \qquad \text{(S4)}$$

After the removal of laser, $Q_{NP} + Q_{sys} = 0$, Eq. S1 can be converted to

$$\sum_i M_i C_i \frac{dT}{dt} = -Q_{out} = -hS(T - T_{surr}) \qquad \text{(S5)}$$

$$dt = \frac{\sum_i M_i C_i}{hS} \frac{dT}{(T - T_{surr})} \qquad \text{(S6)}$$

$$t = -\frac{\sum_i M_i C_i}{hS} \ln \frac{T - T_{surr}}{(T_{max} - T_{surr})} \qquad \text{(S7)}$$

A system time constant τ_s can be defined as

$$\tau_s = -\frac{\sum_i M_i C_i}{hS} \qquad \text{(S8)}$$

and θ is introduced for substitution,

$$\theta = \frac{T - T_{surr}}{(T_{max} - T_{surr})} \qquad \text{(S9)}$$

which transforms Eqs. 8 and 9 into:

$$\mathrm{t} = -\tau_s \ln \theta \qquad \text{(S10)}$$

Since Q_{sys} can be calculated based on

$$Q_{sys} = hS\left(T_{max,H_2O} - T_{surr}\right) \qquad \text{(S11)}$$

Eq. S4 can be expressed as

$$Q_{NP} = I(1 - 10^{-A_{808}})\eta = hS(T_{max} - T_{max,H_2O}) \quad (S12)$$

$$hS = -\frac{\sum_i M_i C_i}{\tau_s} \quad (S13)$$

where τ_s is equal to 203.2 s, m is3.0 g and c is 4.2 J g^{-1}, hS can be calculated as 0.04466 W/°C. Substituting I = 2.0 W, A_{808} = 0.693, $T_{max} - T_{surr}$ = 22.4 °C into Eq. S12, the photothermal conversion efficiency of FPPI NPs can be determined as 51.53%.

S1.4 Quantification of Drug Loading Capacity and Encapsulation Efficiency

The calibration curves of ICG optical absorbance intensity at 780 nm was determined by measuring the corresponding vis-NIR spectrum of ICG. The discarded solution were retained and used for calculating ICG loading capacity (LC) and encapsulation efficiency (EE) based on the calibration curve and according to the following equations,

LC (%) = (weight of devoted ICG-weight of ICG in discarded solution) / (Weight of FPPI NPs) × 100%

EE (%) = (weight of devoted ICG-weight of ICG in discarded solution) / (Weight of devoted ICG) × 100%

S1.5 Cellular Uptake and Internalization

Cellular uptake and internalization of NPs were investigated using fluorescence microscopy. Briefly, HeLa cells were seeded in a 12-well cell culture plate at a density of 2×10^5 cells per well overnight. Afterwards, 1 mL of old medium in each well was discarded and replenished with medium containing free ICG or FPPI NPs at an equivalent concentration of 6 µg mL^{-1}. After further incubation for 2 or 4 h, the cells were stained with DAPI (1 µg mL^{-1}) for 1 min and rinsed with 1×PBS three times. The cells were observed under a fluorescent microscope (IX73, Olympus).

S1.6 Tumor Models *in vivo*

All the animal experiments were carried out in compliance with the National Guide for Care and Use of Laboratory Animals and approved by the Institutional Animal Care and Use Committee (IACUC) of Southwest University. BALB/c nude female mice were obtained from Chengdu Dossy Experimental Animals Co. Ltd. Tumors were induced by subcutaneous injection of 1×10^7 murine breast carcinoma 4T1 cells dispersed in 100 µL of PBS buffer. The tumor volume was monitored post-injection and calculated based on Eq. 14. When the tumor volume exceeded 100 mm^3, the tumor-bearing mice were used for evaluating the combinatorial tumor therapy by PDT/PTT *in vivo*.

$$\text{Tumor volume} = (\text{Tumor length}) \times (\text{Tumor width})^2 \times 0.5 \quad (S14)$$

S1.7 Evaluation of Biocompatibility

Human umbilical vein endothelial cells (HUVECs) were used to evaluate the biocompatibility of FPPI NPs *in vitro*. Firstly, HUVECs with initial seeding density at 1×10^4 per well were cultured in a 96-well cell culture plate at 37 °C for 24 h. Then, old medium in each well was replaced with a fresh medium containing gradient concentrations of FPPI NPs (0-200 μg mL^{-1}). After 24 h of incubation, cell viability was measured according to a standard MTT assay. Hemolysis assay was performed using the blood sample collected from the lateral tail vein of KM mice. Afterwards, mouse whole blood was centrifuged at 3000 rpm for 5 min, and the obtained precipitate was washed four times with 1×PBS to harvest erythrocytes. Then, 0.25 mL of 4% erythrocytes (v/v) was mixed with 0.25 mL of FPPI NPs dispersed in 1×PBS at various concentrations (80, 120, 160, and 200 μg mL^{-1}) and co-incubated at 37 °C for 4 h. Erythrocyte suspensions in DI water and 1×PBS served as the positive and negative control groups, respectively. The samples were centrifuged at 12,000 rpm for 10 min, and the optical absorbance intensity of the supernatants was measured at 570 nm.

In vivo biocompatibility of FPPI NPs was also explored by histological analysis. Normal BALB/c mice were injected with FPPI NP suspension at the ICG concentration of 8 μg mL^{-1}. Then, the major organs, including heart, liver, spleen, lung and kidney, were excised at day 14 post-injection. The obtained organs were fixed with 10% formalin and then sliced into paraffin embedded sections. After standard hematoxylin and eosin (H&E) staining, systemic toxicity of FPPI NPs were examined under a fluorescent microscope.

S1.8 Statistical Analysis

Statistical analyses were conducted using one-way analysis of variance (ANOVA) provided by OriginPro 9.0 software (OriginLab, MA, USA). A *p*-value less than 0.05 (*$p<0.05$, n = 4) was considered as statistically significant.

S2 Supplementary Figures

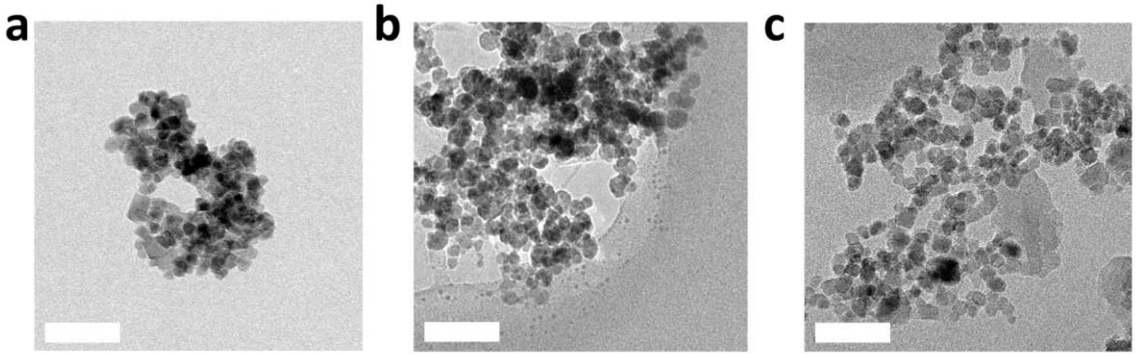

Fig. S1 TEM images of as-synthesized **a** Fe_3O_4 NPs, **b** Fe_3O_4@PB NPs and **c** Fe_3O_4@PB/PEI NPs (scale bars: 50 nm)

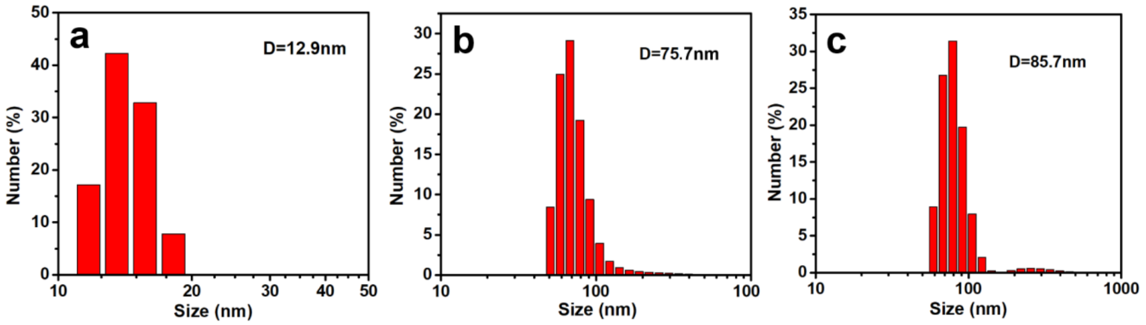

Fig. S2 Size distribution of **a** Fe_3O_4 NPs, **b** Fe_3O_4@PB NPs and **c** Fe_3O_4@PB/PEI NPs measured by dynamic light scattering

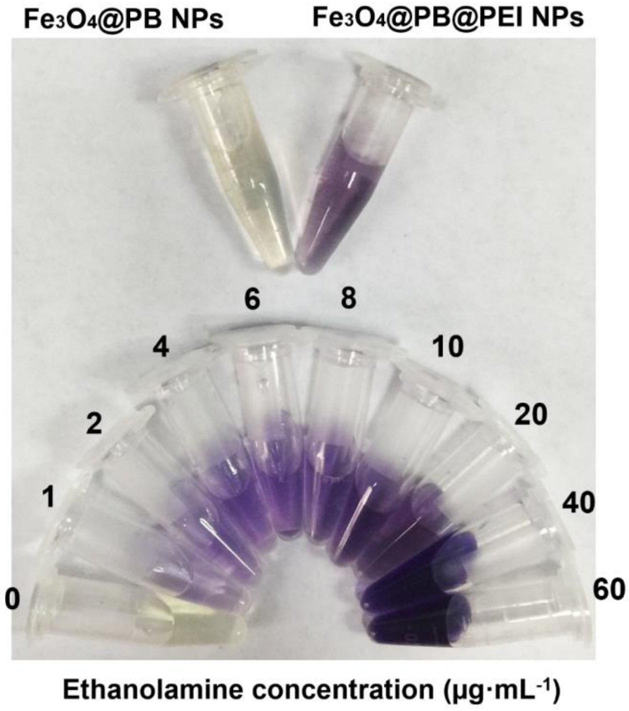

Fig. S3 Photographs of various samples after reaction with ninhydrin reagent. The optical absorbance at 570 nm is positively correlated to the concentration of ethanolamine

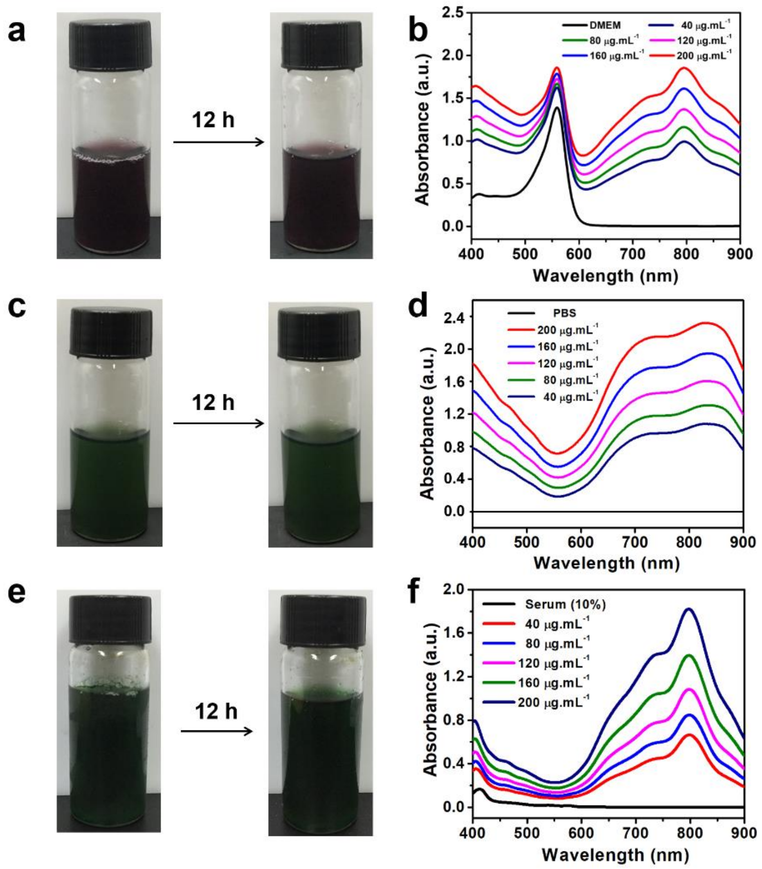

Fig. S4 Photographs of FPPI NPs dispersed in **a** DMEM, **c** 1×PBS or **e** 10% FBS; Vis-NIR absorbance spectra of the FPPI NP dispersions in **b** DMEM, **d** 1×PBS or **f** 10% FBS at various concentrations

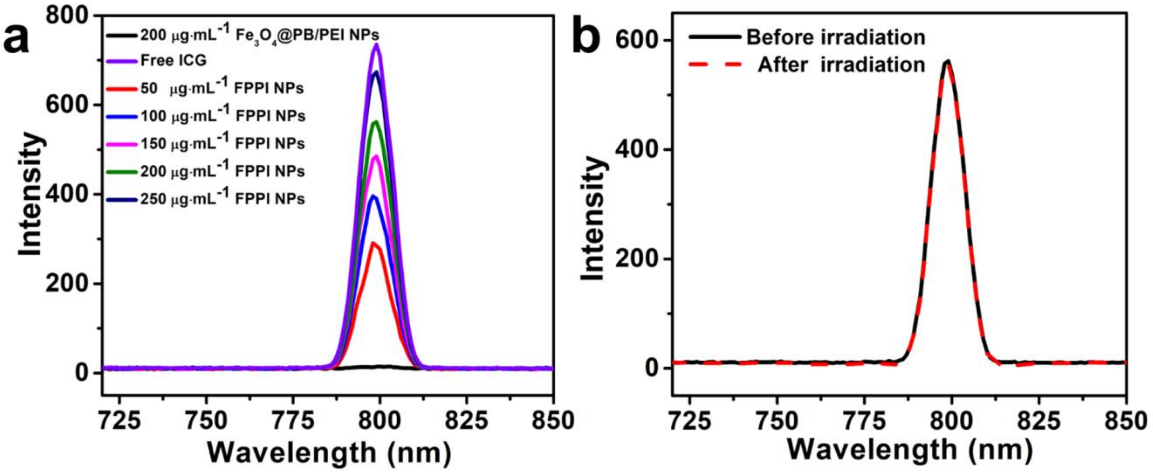

Fig. S5 a Fluorescence spectra of NP dispersions at various concentrations (E_m: 765nm); **b** fluorescence spectra of FPPI aqueous dispersion (200 μg mL^{-1}) before and after NIR irradiation (2 W cm^{-2}, 5 min)

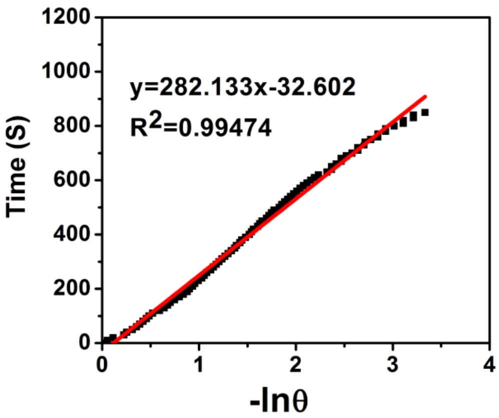

Fig. S6 Fitting curve of the time versus -ln(θ) derived from the cooling period of Fig. 3e

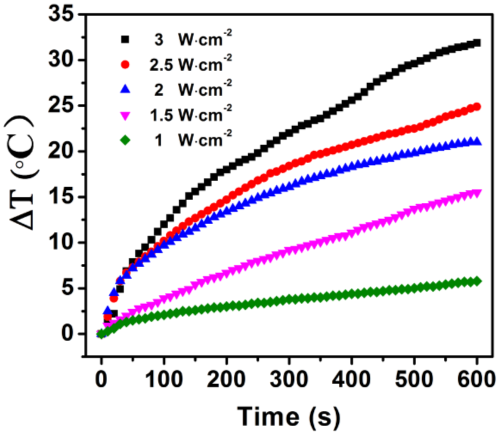

Fig. S7 Temperature variation of FPPI NP aqueous dispersion (200 μg mL^{-1}) irradiated by the NIR laser with various output power density

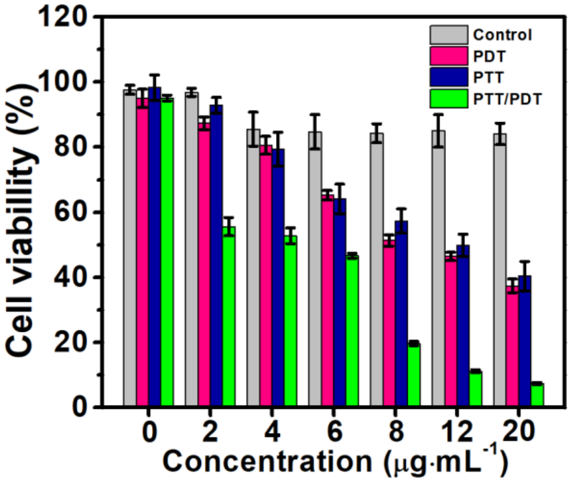

Fig. S8 Relative viability of HeLa cells incubated under different concentration of FPPI NPs subject to individual or combination treatments

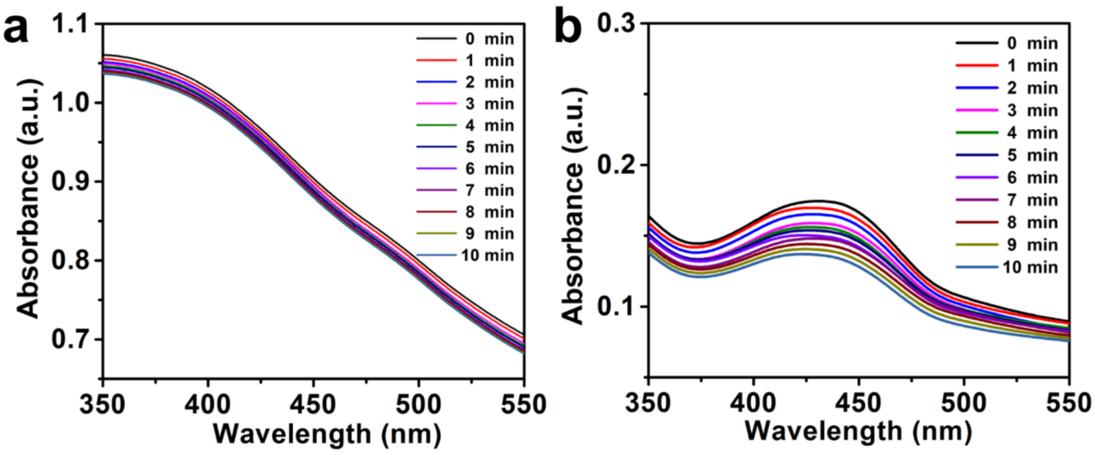

Fig. S9 Decay of DPBF absorbance at the presence of **a** Fe_3O_4@PB/PEI NPs or **b** free ICG under NIR laser irradiation (808nm, 2 W cm^{-2}) for 1~10 min

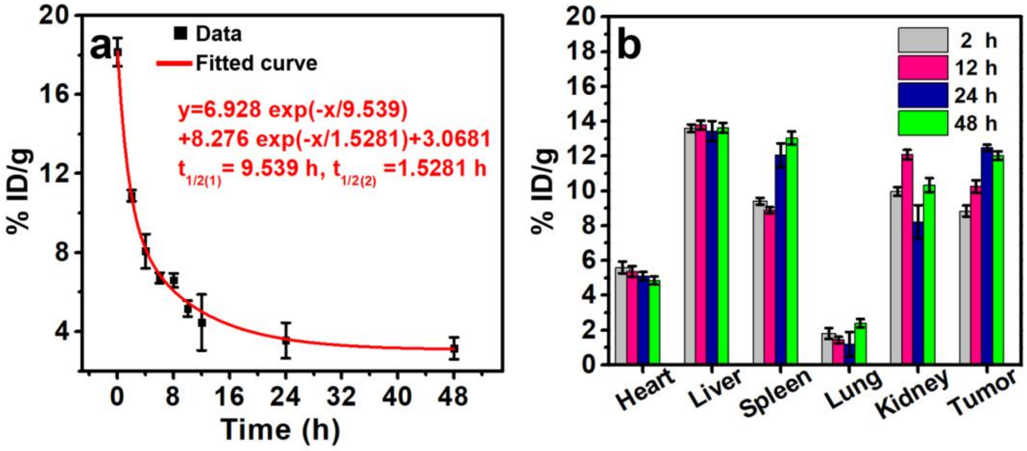

Fig. S10 a *In vivo* blood pharmacokinetics over 24 h after intravenous injection of FPPI NPs; **b** biodistribution of FPPI NPs in major organs at 24 h post-injection

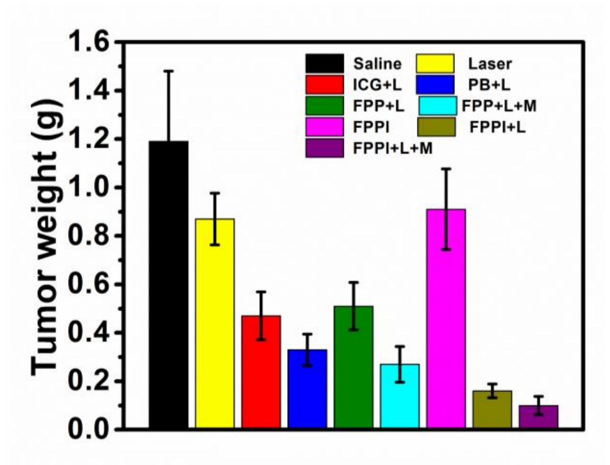

Fig. S11 Weight of the excised tumors in each group at day 14 post-injection

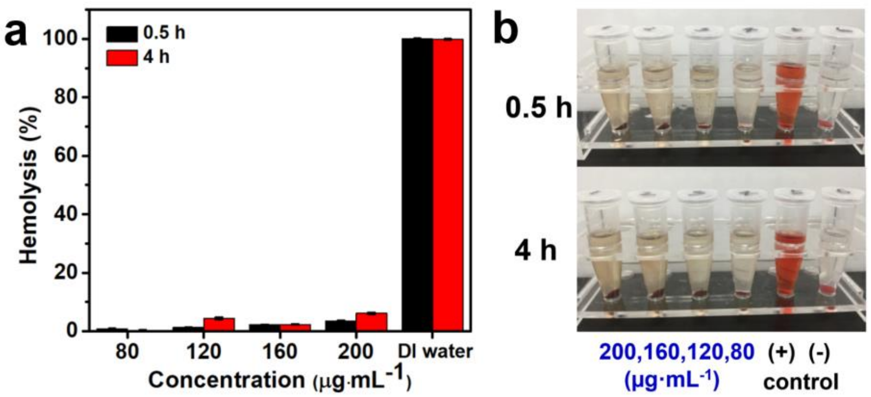

Fig. S12 a Hemolysis rate of RBCs incubated with FPPI NPs at various concentrations; **b** images of RBC suspensions co-incubated with FPPI NPs under various concentrations (positive control: RBCs in DI water; negative control: RBCs in 1×PBS)

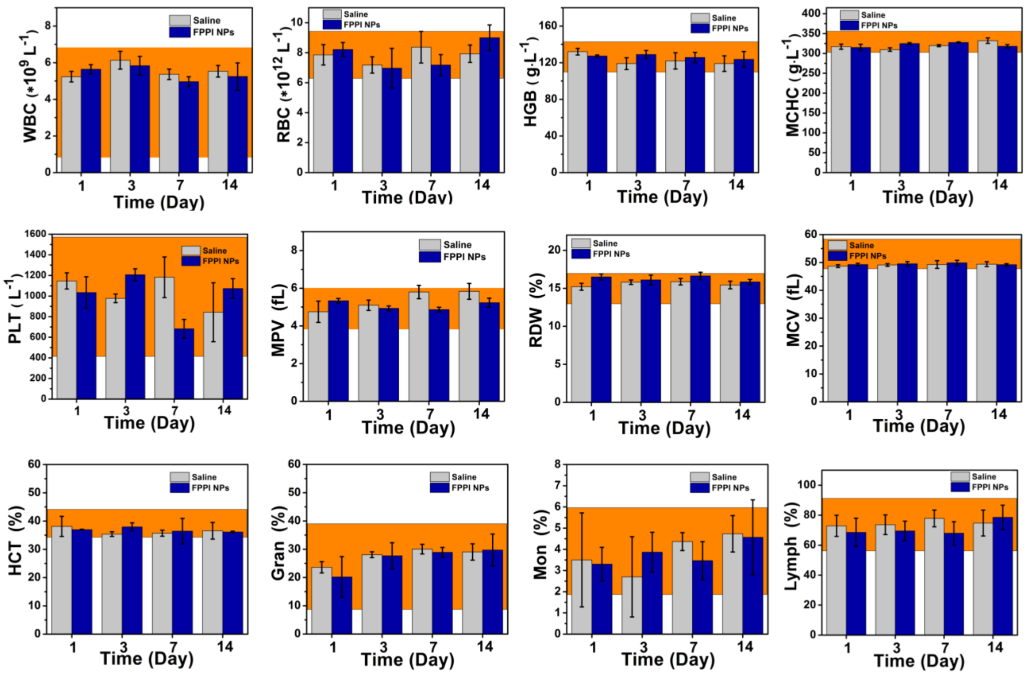

Fig. S13 Complete blood count of health female KM mice. The mice were pre-injected with saline or FPPI NPs. Reference ranges of hematology data of healthy female KM mice (orange hatched area) were obtained from Chongqing Tengxin biotechnology Co. LTD.

Reference

[S1] D.K. Roper, W. Ahn, M. Hoepfner, Microscale heat transfer transduced by surface plasmon resonant gold nanoparticles. J. Phys. Chem. C **111**(9), 3636-3641 (2007). http://doi.org/10.1021/jp064341w
